# Supplementary material for: Hot spot prediction in protein-protein interactions by an ensemble system
Source: BMC Syst Biol. 2018 Dec 31;12(Suppl 9):132. doi: 10.1186/s12918-018-0665-8 (PMC6311905; doi:10.1186/s12918-018-0665-8)
Supplement: Supplementary file 2 — All relevant MATLAB code. A simple Matlab implement of our predictor. (ZIP 19,742 kb) [file 12918_2018_665_MOESM2_ESM.zip › Additional file 2/Read ASA/ASA website/dbMPIKT/4YFDB.html]

 NetSurfP - Protein Surface Accessibility and Secondary Structure Predictions

  

|  |  |  |
| --- | --- | --- |
|  |  | NetSurfP - Protein Surface Accessibility and Secondary Structure Predictions  Technical University of Denmark |

  


---

```
# For publication of results, please cite:
# A generic method for assignment of reliability scores applied to solvent accessibility predictions.
# Bent Petersen, Thomas Nordahl Petersen, Pernille Andersen, Morten Nielsen and Claus Lundegaard
# BMC Structural Biology 2009, 9:51 doi:10.1186/1472-6807-9-51
#
# Column 1: Class assignment - B for buried or E for Exposed - Threshold: 25% exposure, but not based on RSA
# Column 2: Amino acid
# Column 3: Sequence name
# Column 4: Amino acid number
# Column 5: Relative Surface Accessibility - RSA
# Column 6: Absolute Surface Accessibility
# Column 7: Z-fit score for RSA prediction
# Column 8: Probability for Alpha-Helix
# Column 9: Probability for Beta-strand
# Column 10: Probability for Coil
E S  4YFDB                  1    0.766  89.799  -0.686   0.003   0.003   0.994
E E  4YFDB                  2    0.517  90.267  -1.072   0.018   0.047   0.935
E R  4YFDB                  3    0.603 137.995   1.688   0.053   0.043   0.903
B C  4YFDB                  4    0.084  11.808  -0.424   0.052   0.084   0.864
E D  4YFDB                  5    0.543  78.318   0.805   0.113   0.087   0.800
E D  4YFDB                  6    0.500  72.050   0.547   0.056   0.142   0.802
B W  4YFDB                  7    0.209  50.337   0.952   0.056   0.142   0.802
E G  4YFDB                  8    0.495  38.949  -0.344   0.056   0.142   0.802
E L  4YFDB                  9    0.367  67.124  -0.035   0.019   0.141   0.840
E D  4YFDB                 10    0.578  83.304   0.378   0.018   0.088   0.893
E T  4YFDB                 11    0.374  51.874  -0.072   0.018   0.047   0.935
E M  4YFDB                 12    0.346  69.235  -0.780   0.018   0.047   0.935
E R  4YFDB                 13    0.583 133.599  -0.041   0.018   0.088   0.893
E Q  4YFDB                 14    0.553  98.766   0.462   0.005   0.336   0.660
B I  4YFDB                 15    0.110  20.294   1.065   0.002   0.816   0.182
E Q  4YFDB                 16    0.453  80.906   1.378   0.001   0.959   0.040
B V  4YFDB                 17    0.060   9.176   0.368   0.001   0.959   0.040
E F  4YFDB                 18    0.377  75.724   1.374   0.002   0.816   0.182
E E  4YFDB                 19    0.465  81.270   0.631   0.004   0.197   0.799
E D  4YFDB                 20    0.728 104.876  -1.065   0.005   0.015   0.979
E E  4YFDB                 21    0.447  78.108   1.050   0.005   0.015   0.979
E P  4YFDB                 22    0.529  75.108   1.245   0.004   0.420   0.576
B A  4YFDB                 23    0.042   4.661   0.395   0.001   0.900   0.099
E R  4YFDB                 24    0.330  75.570   1.809   0.001   0.959   0.040
B I  4YFDB                 25    0.034   6.234   0.473   0.001   0.959   0.040
E K  4YFDB                 26    0.371  76.335   1.643   0.001   0.959   0.040
B C  4YFDB                 27    0.020   2.766   0.933   0.001   0.900   0.099
E P  4YFDB                 28    0.428  60.747   1.296   0.001   0.900   0.099
B L  4YFDB                 29    0.076  13.952  -1.596   0.003   0.718   0.279
E F  4YFDB                 30    0.417  83.712  -0.092   0.005   0.336   0.660
E E  4YFDB                 31    0.637 111.319  -2.259   0.018   0.088   0.893
E H  4YFDB                 32    0.587 106.812  -1.484   0.019   0.141   0.840
E F  4YFDB                 33    0.439  88.067  -1.322   0.020   0.205   0.775
B L  4YFDB                 34    0.266  48.760  -0.912   0.022   0.552   0.426
E K  4YFDB                 35    0.452  93.079  -0.172   0.023   0.655   0.322
B Y  4YFDB                 36    0.183  39.128  -0.521   0.023   0.655   0.322
E N  4YFDB                 37    0.393  57.550  -0.827   0.022   0.552   0.426
B Y  4YFDB                 38    0.194  41.458  -1.192   0.022   0.552   0.426
E S  4YFDB                 39    0.411  48.193  -1.085   0.021   0.451   0.528
B T  4YFDB                 40    0.312  43.205  -2.132   0.022   0.359   0.619
E A  4YFDB                 41    0.526  57.987  -1.731   0.020   0.205   0.775
E H  4YFDB                 42    0.539  98.099  -2.709   0.004   0.085   0.910
E S  4YFDB                 43    0.494  57.885  -1.110   0.005   0.015   0.979
E S  4YFDB                 44    0.399  46.704  -1.458   0.005   0.045   0.951
E G  4YFDB                 45    0.515  40.570   0.077   0.004   0.138   0.858
B L  4YFDB                 46    0.075  13.696  -0.667   0.005   0.336   0.660
E T  4YFDB                 47    0.360  49.974   1.219   0.002   0.816   0.182
B L  4YFDB                 48    0.029   5.273   0.515   0.001   0.959   0.040
E I  4YFDB                 49    0.226  41.866   1.686   0.001   0.959   0.040
B W  4YFDB                 50    0.024   5.868   0.874   0.001   0.959   0.040
B Y  4YFDB                 51    0.209  44.685   0.910   0.001   0.959   0.040
B W  4YFDB                 52    0.141  33.935   0.892   0.001   0.900   0.099
E T  4YFDB                 53    0.428  59.364   0.277   0.020   0.205   0.775
E R  4YFDB                 54    0.626 143.262  -1.902   0.018   0.047   0.935
E Q  4YFDB                 55    0.653 116.608  -0.456   0.005   0.336   0.660
E D  4YFDB                 56    0.660  95.106  -0.819   0.005   0.336   0.660
E R  4YFDB                 57    0.307  70.303  -0.439   0.020   0.205   0.775
E D  4YFDB                 58    0.501  72.266  -1.035   0.020   0.205   0.775
E L  4YFDB                 59    0.395  72.251  -0.947   0.021   0.279   0.699
E E  4YFDB                 60    0.512  89.394  -1.837   0.021   0.279   0.699
E E  4YFDB                 61    0.473  82.616  -0.971   0.021   0.451   0.528
E P  4YFDB                 62    0.493  69.928  -0.842   0.023   0.655   0.322
B I  4YFDB                 63    0.229  42.402  -0.640   0.021   0.756   0.223
E N  4YFDB                 64    0.459  67.139  -0.831   0.021   0.756   0.223
B F  4YFDB                 65    0.286  57.320  -1.115   0.021   0.756   0.223
E R  4YFDB                 66    0.448 102.592  -1.028   0.004   0.616   0.381
E L  4YFDB                 67    0.316  57.841  -0.981   0.005   0.262   0.733
E P  4YFDB                 68    0.630  89.369  -1.305   0.004   0.138   0.858
E E  4YFDB                 69    0.562  98.181  -1.609   0.004   0.197   0.799
B N  4YFDB                 70    0.245  35.912  -1.493   0.004   0.514   0.481
E R  4YFDB                 71    0.308  70.486  -0.083   0.001   0.900   0.099
B I  4YFDB                 72    0.144  26.603  -0.486   0.001   0.959   0.040
E S  4YFDB                 73    0.289  33.824  -0.045   0.001   0.959   0.040
E K  4YFDB                 74    0.296  60.825  -0.526   0.002   0.816   0.182
E E  4YFDB                 75    0.508  88.817  -1.388   0.005   0.336   0.660
E K  4YFDB                 76    0.616 126.773  -0.994   0.005   0.045   0.951
B D  4YFDB                 77    0.188  27.105  -0.988   0.004   0.197   0.799
B V  4YFDB                 78    0.147  22.517   0.623   0.001   0.900   0.099
B L  4YFDB                 79    0.018   3.259   1.027   0.001   0.959   0.040
B W  4YFDB                 80    0.194  46.753   1.594   0.006   0.962   0.032
B F  4YFDB                 81    0.022   4.456   0.687   0.001   0.900   0.099
E R  4YFDB                 82    0.390  89.379   1.402   0.002   0.816   0.182
E P  4YFDB                 83    0.513  72.766   0.730   0.004   0.514   0.481
B T  4YFDB                 84    0.070   9.764   0.168   0.005   0.262   0.733
E L  4YFDB                 85    0.460  84.171   1.295   0.004   0.420   0.576
E L  4YFDB                 86    0.517  94.718   0.055   0.004   0.420   0.576
E N  4YFDB                 87    0.611  89.480  -0.572   0.004   0.138   0.858
B D  4YFDB                 88    0.244  35.232  -0.801   0.005   0.045   0.951
E T  4YFDB                 89    0.519  72.041  -0.324   0.004   0.085   0.910
B G  4YFDB                 90    0.187  14.701  -1.483   0.004   0.616   0.381
E N  4YFDB                 91    0.361  52.850   1.758   0.001   0.900   0.099
B Y  4YFDB                 92    0.044   9.488   0.637   0.001   0.959   0.040
B T  4YFDB                 93    0.223  30.875   1.865   0.001   0.959   0.040
B C  4YFDB                 94    0.013   1.867   1.564   0.001   0.959   0.040
B M  4YFDB                 95    0.214  42.841   1.697   0.001   0.959   0.040
B L  4YFDB                 96    0.035   6.427  -0.496   0.001   0.959   0.040
E R  4YFDB                 97    0.383  87.730   0.496   0.001   0.959   0.040
B N  4YFDB                 98    0.134  19.647  -0.517   0.004   0.616   0.381
E T  4YFDB                 99    0.624  86.604  -0.709   0.005   0.045   0.951
E T  4YFDB                100    0.554  76.909  -1.178   0.004   0.197   0.799
E Y  4YFDB                101    0.444  94.947  -1.166   0.004   0.514   0.481
E C  4YFDB                102    0.438  61.551  -0.821   0.002   0.816   0.182
E S  4YFDB                103    0.342  40.047  -0.575   0.001   0.900   0.099
E K  4YFDB                104    0.457  94.067   0.393   0.001   0.959   0.040
B V  4YFDB                105    0.132  20.258   0.701   0.001   0.959   0.040
E A  4YFDB                106    0.344  37.865   0.664   0.001   0.959   0.040
B F  4YFDB                107    0.039   7.888  -0.244   0.001   0.959   0.040
E P  4YFDB                108    0.275  39.022   1.381   0.001   0.959   0.040
B L  4YFDB                109    0.021   3.808   1.004   0.001   0.959   0.040
E E  4YFDB                110    0.278  48.602   2.075   0.001   0.959   0.040
B V  4YFDB                111    0.030   4.657   0.243   0.001   0.959   0.040
E V  4YFDB                112    0.301  46.294   0.847   0.001   0.900   0.099
E Q  4YFDB                113    0.382  68.297  -0.020   0.003   0.718   0.279
E K  4YFDB                114    0.422  86.888   0.199   0.020   0.205   0.775
E D  4YFDB                115    0.443  63.822  -1.064   0.018   0.047   0.935
E S  4YFDB                116    0.512  60.065  -1.577   0.018   0.088   0.893
E C  4YFDB                117    0.485  68.122  -1.915   0.018   0.088   0.893
E F  4YFDB                118    0.420  84.274  -1.181   0.018   0.088   0.893
E N  4YFDB                119    0.350  51.255  -1.559   0.018   0.088   0.893
E S  4YFDB                120    0.586  68.691  -1.405   0.020   0.205   0.775
E A  4YFDB                121    0.362  39.925  -1.411   0.021   0.279   0.699
E M  4YFDB                122    0.351  70.315  -0.880   0.020   0.205   0.775
E R  4YFDB                123    0.471 107.882  -0.843   0.020   0.205   0.775
E F  4YFDB                124    0.414  83.070  -0.723   0.018   0.088   0.893
B P  4YFDB                125    0.289  41.052  -1.546   0.018   0.088   0.893
E V  4YFDB                126    0.528  81.092  -0.813   0.019   0.141   0.840
E H  4YFDB                127    0.525  95.425  -0.915   0.021   0.451   0.528
E K  4YFDB                128    0.310  63.746   0.129   0.021   0.756   0.223
E M  4YFDB                129    0.454  90.845   0.127   0.018   0.846   0.136
B Y  4YFDB                130    0.160  34.299  -0.461   0.011   0.918   0.071
E I  4YFDB                131    0.423  78.237   0.093   0.002   0.816   0.182
E E  4YFDB                132    0.451  78.720  -0.187   0.004   0.420   0.576
E H  4YFDB                133    0.676 122.928  -1.019   0.004   0.085   0.910
E G  4YFDB                134    0.572  44.985   0.007   0.004   0.085   0.910
E I  4YFDB                135    0.571 105.598   0.178   0.004   0.420   0.576
B H  4YFDB                136    0.092  16.698   0.082   0.001   0.900   0.099
E K  4YFDB                137    0.368  75.615   1.313   0.001   0.959   0.040
B I  4YFDB                138    0.041   7.567   0.084   0.001   0.959   0.040
E T  4YFDB                139    0.364  50.556   0.800   0.001   0.959   0.040
B C  4YFDB                140    0.034   4.816  -0.427   0.002   0.816   0.182
E P  4YFDB                141    0.377  53.539  -0.304   0.004   0.514   0.481
B N  4YFDB                142    0.344  50.318  -1.226   0.004   0.514   0.481
B V  4YFDB                143    0.198  30.356  -0.443   0.004   0.616   0.381
E D  4YFDB                144    0.453  65.220  -0.724   0.021   0.451   0.528
B G  4YFDB                145    0.189  14.890  -1.041   0.023   0.655   0.322
E Y  4YFDB                146    0.429  91.741  -0.136   0.021   0.756   0.223
B F  4YFDB                147    0.123  24.606  -1.405   0.004   0.616   0.381
E P  4YFDB                148    0.523  74.242  -0.535   0.005   0.336   0.660
E S  4YFDB                149    0.496  58.119  -2.338   0.004   0.085   0.910
E S  4YFDB                150    0.416  48.720  -0.434   0.005   0.045   0.951
B V  4YFDB                151    0.276  42.437  -0.537   0.005   0.045   0.951
E K  4YFDB                152    0.494 101.616   0.899   0.004   0.138   0.858
B P  4YFDB                153    0.098  13.864  -0.070   0.005   0.336   0.660
E S  4YFDB                154    0.399  46.786   1.326   0.002   0.816   0.182
B V  4YFDB                155    0.038   5.841   0.237   0.001   0.959   0.040
E T  4YFDB                156    0.295  40.861   1.891   0.001   0.959   0.040
B W  4YFDB                157    0.033   7.961   0.202   0.001   0.959   0.040
B Y  4YFDB                158    0.229  49.023   0.959   0.001   0.959   0.040
B K  4YFDB                159    0.235  48.339   0.450   0.003   0.718   0.279
E G  4YFDB                160    0.599  47.173  -0.703   0.018   0.047   0.935
E C  4YFDB                161    0.602  84.465  -1.803   0.005   0.045   0.951
E T  4YFDB                162    0.551  76.396   0.380   0.004   0.616   0.381
E E  4YFDB                163    0.498  87.088   0.415   0.004   0.616   0.381
B I  4YFDB                164    0.089  16.539   0.092   0.021   0.279   0.699
E V  4YFDB                165    0.527  81.061   0.851   0.004   0.138   0.858
E D  4YFDB                166    0.704 101.461  -1.019   0.004   0.085   0.910
E F  4YFDB                167    0.651 130.636  -1.430   0.005   0.045   0.951
E H  4YFDB                168    0.392  71.268  -1.298   0.004   0.197   0.799
E N  4YFDB                169    0.338  49.527  -0.302   0.002   0.816   0.182
B V  4YFDB                170    0.266  40.823  -0.496   0.001   0.900   0.099
E L  4YFDB                171    0.318  58.226   0.085   0.001   0.900   0.099
E P  4YFDB                172    0.323  45.848  -0.854   0.003   0.718   0.279
E E  4YFDB                173    0.625 109.153  -1.214   0.005   0.262   0.733
E G  4YFDB                174    0.670  52.729  -0.777   0.005   0.045   0.951
B M  4YFDB                175    0.253  50.545  -0.432   0.004   0.138   0.858
E N  4YFDB                176    0.282  41.270   1.197   0.002   0.816   0.182
B L  4YFDB                177    0.023   4.248   0.773   0.001   0.959   0.040
E S  4YFDB                178    0.238  27.905   1.803   0.001   0.959   0.040
B F  4YFDB                179    0.029   5.720   0.451   0.001   0.959   0.040
E F  4YFDB                180    0.408  81.886   1.493   0.001   0.900   0.099
E I  4YFDB                181    0.519  95.941   0.585   0.004   0.616   0.381
B P  4YFDB                182    0.076  10.799  -0.029   0.005   0.336   0.660
E L  4YFDB                183    0.408  74.778   1.087   0.005   0.336   0.660
E V  4YFDB                184    0.564  86.764   0.447   0.005   0.262   0.733
E S  4YFDB                185    0.654  76.649  -0.093   0.004   0.085   0.910
B N  4YFDB                186    0.222  32.486  -0.955   0.005   0.045   0.951
E N  4YFDB                187    0.512  74.913  -0.158   0.004   0.138   0.858
B G  4YFDB                188    0.153  12.010  -1.662   0.003   0.718   0.279
E N  4YFDB                189    0.403  58.941   1.834   0.001   0.900   0.099
B Y  4YFDB                190    0.047  10.065   0.699   0.001   0.959   0.040
B T  4YFDB                191    0.243  33.649   1.852   0.001   0.959   0.040
B C  4YFDB                192    0.013   1.895   1.636   0.001   0.959   0.040
E V  4YFDB                193    0.230  35.412   1.994   0.001   0.959   0.040
B V  4YFDB                194    0.025   3.796   0.632   0.001   0.959   0.040
E T  4YFDB                195    0.316  43.829   0.836   0.001   0.959   0.040
B Y  4YFDB                196    0.098  20.857   0.431   0.001   0.900   0.099
E P  4YFDB                197    0.461  65.458  -0.165   0.005   0.336   0.660
E E  4YFDB                198    0.506  88.381  -1.312   0.004   0.138   0.858
E N  4YFDB                199    0.619  90.622  -2.303   0.004   0.085   0.910
E G  4YFDB                200    0.517  40.696  -2.226   0.004   0.138   0.858
E R  4YFDB                201    0.527 120.591  -0.829   0.004   0.514   0.481
E L  4YFDB                202    0.481  88.016  -0.706   0.004   0.514   0.481
B F  4YFDB                203    0.288  57.842  -0.218   0.004   0.616   0.381
E H  4YFDB                204    0.501  91.041  -0.299   0.002   0.816   0.182
B L  4YFDB                205    0.112  20.471  -0.653   0.001   0.900   0.099
E T  4YFDB                206    0.385  53.427   0.290   0.001   0.900   0.099
B R  4YFDB                207    0.155  35.381   0.186   0.001   0.900   0.099
E T  4YFDB                208    0.297  41.138   0.513   0.001   0.959   0.040
B V  4YFDB                209    0.029   4.427   0.296   0.001   0.959   0.040
E T  4YFDB                210    0.289  40.084   1.530   0.001   0.959   0.040
B V  4YFDB                211    0.022   3.428   0.984   0.001   0.959   0.040
E K  4YFDB                212    0.286  58.871   1.943   0.001   0.959   0.040
B V  4YFDB                213    0.035   5.380  -0.487   0.001   0.900   0.099
E V  4YFDB                214    0.403  61.941   0.820   0.002   0.816   0.182
E G  4YFDB                215    0.321  25.286  -0.590   0.004   0.514   0.481
E S  4YFDB                216    0.434  50.841  -0.097   0.020   0.205   0.775
E P  4YFDB                217    0.497  70.496  -1.282   0.018   0.088   0.893
E K  4YFDB                218    0.595 122.289  -1.122   0.020   0.205   0.775
E D  4YFDB                219    0.366  52.712  -1.023   0.022   0.359   0.619
E A  4YFDB                220    0.462  50.956  -0.630   0.022   0.359   0.619
E L  4YFDB                221    0.358  65.513  -0.265   0.020   0.205   0.775
E P  4YFDB                222    0.278  39.448  -0.081   0.018   0.088   0.893
B P  4YFDB                223    0.207  29.444  -1.258   0.020   0.205   0.775
E Q  4YFDB                224    0.441  78.745  -0.313   0.022   0.552   0.426
B I  4YFDB                225    0.177  32.671  -1.729   0.023   0.655   0.322
E Y  4YFDB                226    0.335  71.675  -1.146   0.021   0.451   0.528
E S  4YFDB                227    0.356  41.735  -0.701   0.020   0.205   0.775
E P  4YFDB                228    0.371  52.645  -0.605   0.018   0.047   0.935
E N  4YFDB                229    0.500  73.215  -0.896   0.018   0.047   0.935
E D  4YFDB                230    0.622  89.587  -1.224   0.018   0.088   0.893
E R  4YFDB                231    0.555 127.026  -1.357   0.020   0.205   0.775
E V  4YFDB                232    0.378  58.114  -0.543   0.004   0.514   0.481
E V  4YFDB                233    0.412  63.340  -0.351   0.002   0.816   0.182
B Y  4YFDB                234    0.245  52.399   0.813   0.011   0.918   0.071
E E  4YFDB                235    0.459  80.170   0.037   0.001   0.900   0.099
B K  4YFDB                236    0.090  18.451   0.208   0.002   0.816   0.182
E E  4YFDB                237    0.480  83.839   0.331   0.004   0.616   0.381
E P  4YFDB                238    0.460  65.246   0.032   0.004   0.197   0.799
E G  4YFDB                239    0.641  50.415  -1.389   0.005   0.015   0.979
E E  4YFDB                240    0.488  85.323   0.247   0.004   0.085   0.910
E E  4YFDB                241    0.558  97.518   1.171   0.004   0.616   0.381
B L  4YFDB                242    0.045   8.203   0.179   0.001   0.900   0.099
E V  4YFDB                243    0.385  59.159   1.900   0.001   0.959   0.040
B I  4YFDB                244    0.034   6.272   0.725   0.001   0.959   0.040
E P  4YFDB                245    0.339  48.118   1.862   0.001   0.900   0.099
B C  4YFDB                246    0.014   1.966   1.393   0.001   0.900   0.099
E K  4YFDB                247    0.339  69.712   2.118   0.001   0.959   0.040
B V  4YFDB                248    0.032   4.980  -0.639   0.001   0.959   0.040
E Y  4YFDB                249    0.343  73.278   0.601   0.001   0.900   0.099
B F  4YFDB                250    0.285  57.240  -0.511   0.003   0.718   0.279
E S  4YFDB                251    0.387  45.415  -0.754   0.005   0.262   0.733
E F  4YFDB                252    0.502 100.671  -1.625   0.004   0.085   0.910
E I  4YFDB                253    0.636 117.734  -1.941   0.005   0.045   0.951
E M  4YFDB                254    0.356  71.176  -2.489   0.005   0.015   0.979
E D  4YFDB                255    0.533  76.820  -0.279   0.005   0.015   0.979
B S  4YFDB                256    0.154  18.084  -0.937   0.004   0.085   0.910
E H  4YFDB                257    0.449  81.619   0.445   0.004   0.197   0.799
B N  4YFDB                258    0.069  10.116  -0.558   0.004   0.514   0.481
E E  4YFDB                259    0.276  48.287   1.064   0.001   0.900   0.099
B V  4YFDB                260    0.029   4.519   0.565   0.001   0.959   0.040
B W  4YFDB                261    0.198  47.499   1.805   0.001   0.959   0.040
B W  4YFDB                262    0.025   5.940   0.847   0.001   0.959   0.040
B T  4YFDB                263    0.226  31.318   1.309   0.001   0.959   0.040
B I  4YFDB                264    0.185  34.151   1.012   0.002   0.816   0.182
E D  4YFDB                265    0.582  83.909  -0.238   0.018   0.047   0.935
E G  4YFDB                266    0.655  51.564  -2.006   0.018   0.019   0.964
E K  4YFDB                267    0.570 117.167  -0.107   0.004   0.616   0.381
E K  4YFDB                268    0.486  99.991   0.129   0.004   0.616   0.381
B P  4YFDB                269    0.109  15.439  -0.869   0.022   0.359   0.619
E D  4YFDB                270    0.522  75.220  -0.811   0.021   0.279   0.699
E D  4YFDB                271    0.538  77.540  -1.602   0.019   0.141   0.840
E V  4YFDB                272    0.470  72.239  -2.328   0.018   0.047   0.935
E T  4YFDB                273    0.523  72.526  -2.212   0.018   0.088   0.893
E V  4YFDB                274    0.505  77.665  -1.607   0.020   0.205   0.775
E D  4YFDB                275    0.369  53.144  -1.394   0.022   0.359   0.619
B I  4YFDB                276    0.275  50.838  -1.375   0.023   0.655   0.322
E T  4YFDB                277    0.373  51.763  -1.087   0.021   0.756   0.223
B I  4YFDB                278    0.270  49.913  -0.909   0.003   0.718   0.279
E N  4YFDB                279    0.394  57.638  -1.201   0.004   0.616   0.381
E E  4YFDB                280    0.579 101.169  -1.502   0.004   0.420   0.576
E S  4YFDB                281    0.515  60.323  -2.215   0.005   0.336   0.660
E V  4YFDB                282    0.498  76.496  -1.437   0.004   0.514   0.481
E S  4YFDB                283    0.439  51.498  -1.154   0.002   0.816   0.182
E Y  4YFDB                284    0.295  63.084  -0.342   0.001   0.900   0.099
E S  4YFDB                285    0.353  41.325  -0.665   0.001   0.900   0.099
B S  4YFDB                286    0.222  26.065  -1.381   0.001   0.900   0.099
B T  4YFDB                287    0.282  39.044  -1.319   0.003   0.718   0.279
E E  4YFDB                288    0.525  91.805  -1.294   0.005   0.336   0.660
E D  4YFDB                289    0.665  95.798  -2.025   0.004   0.085   0.910
E E  4YFDB                290    0.563  98.374  -2.029   0.004   0.138   0.858
E T  4YFDB                291    0.424  58.753  -0.989   0.004   0.514   0.481
B R  4YFDB                292    0.245  56.036  -0.494   0.002   0.816   0.182
E T  4YFDB                293    0.289  40.029   0.298   0.001   0.900   0.099
B Q  4YFDB                294    0.064  11.430  -0.209   0.001   0.900   0.099
B I  4YFDB                295    0.150  27.787   0.770   0.001   0.900   0.099
B L  4YFDB                296    0.016   2.893   1.393   0.001   0.959   0.040
E S  4YFDB                297    0.211  24.788   1.651   0.001   0.959   0.040
B I  4YFDB                298    0.021   3.922   0.800   0.001   0.959   0.040
E K  4YFDB                299    0.417  85.777   1.340   0.002   0.816   0.182
E K  4YFDB                300    0.530 109.021   0.545   0.004   0.616   0.381
B V  4YFDB                301    0.105  16.108   0.455   0.021   0.451   0.528
E T  4YFDB                302    0.406  56.340   0.909   0.004   0.514   0.481
E P  4YFDB                303    0.553  78.542   0.118   0.004   0.616   0.381
E E  4YFDB                304    0.545  95.142   0.069   0.005   0.336   0.660
E D  4YFDB                305    0.311  44.801  -1.006   0.004   0.197   0.799
E L  4YFDB                306    0.409  74.833  -0.886   0.004   0.085   0.910
E R  4YFDB                307    0.566 129.591  -0.261   0.004   0.138   0.858
B R  4YFDB                308    0.146  33.480  -0.026   0.004   0.616   0.381
E N  4YFDB                309    0.426  62.410   1.314   0.002   0.816   0.182
B Y  4YFDB                310    0.051  10.899   0.915   0.001   0.959   0.040
B V  4YFDB                311    0.264  40.608   1.658   0.001   0.959   0.040
B C  4YFDB                312    0.014   1.980   1.564   0.001   0.959   0.040
E H  4YFDB                313    0.227  41.364   1.934   0.001   0.959   0.040
B A  4YFDB                314    0.036   3.912  -0.826   0.001   0.959   0.040
E R  4YFDB                315    0.355  81.295   0.715   0.001   0.959   0.040
B N  4YFDB                316    0.105  15.416  -0.077   0.003   0.718   0.279
E T  4YFDB                317    0.575  79.808   0.431   0.004   0.197   0.799
E K  4YFDB                318    0.371  76.294  -0.401   0.005   0.336   0.660
E G  4YFDB                319    0.367  28.875  -1.085   0.004   0.616   0.381
E E  4YFDB                320    0.467  81.567   0.136   0.002   0.816   0.182
B A  4YFDB                321    0.234  25.820  -0.610   0.001   0.959   0.040
E E  4YFDB                322    0.452  78.947   0.369   0.001   0.959   0.040
B Q  4YFDB                323    0.129  23.004   0.567   0.001   0.959   0.040
E A  4YFDB                324    0.330  36.322   0.848   0.001   0.959   0.040
B A  4YFDB                325    0.025   2.788   0.457   0.000   0.983   0.017
E K  4YFDB                326    0.283  58.193   2.014   0.000   0.983   0.017
B V  4YFDB                327    0.023   3.550   0.921   0.001   0.959   0.040
E K  4YFDB                328    0.319  65.659   2.116   0.001   0.959   0.040
B Q  4YFDB                329    0.084  14.967   0.223   0.001   0.900   0.099
E K  4YFDB                330    0.476  97.810   1.013   0.002   0.816   0.182
E V  4YFDB                331    0.330  50.782  -0.403   0.021   0.451   0.528
E A  4YFDB                332    0.397  43.760  -0.589   0.019   0.141   0.840
B A  4YFDB                333    0.259  28.520  -0.941   0.018   0.019   0.964
E H  4YFDB                334    0.440  80.036  -0.619   0.018   0.019   0.964
E H  4YFDB                335    0.576 104.756  -0.258   0.018   0.047   0.935
E H  4YFDB                336    0.361  65.757  -0.960   0.018   0.088   0.893
E H  4YFDB                337    0.618 112.432  -0.334   0.019   0.141   0.840
E H  4YFDB                338    0.485  88.258  -1.012   0.018   0.088   0.893
E H  4YFDB                339    0.699 127.057  -2.598   0.003   0.003   0.994

Explain the output.  Go back.
```

---
